# The Origin And Migration Of Primordial Germ Cells In Sturgeons

**DOI:** 10.1371/journal.pone.0086861

**Published:** 2014-02-05

**Authors:** Taiju Saito, Martin Pšenička, Rie Goto, Shinji Adachi, Kunio Inoue, Katsutoshi Arai, Etsuro Yamaha

**Affiliations:** 1 Nanae Fresh Water Laboratory, Field Science Center for Northern Biosphere, Hokkaido University, Nanae, Japan; 2 Laboratory of Aquaculture Genetics & Genomics, Faculty of Fisheries Sciences, Hokkaido University, Hakodate, Japan; 3 Laboratory of Reproductive Physiology, Research Institute of Fish Culture and Hydrobiology, South Bohemian Research Center of Aquaculture and Biodiversity of Hydrocenoses, Faculty of Fisheries and Protection of Waters, University of South Bohemia in Ceske Budejovice, Vodňany, Czech Republic; 4 Laboratory of Aquaculture Biology, Faculty of Fisheries Sciences, Hokkaido University, Hakodate, Japan; 5 Department of Biology, Faculty of Science, Kobe University, Kobe, Japan; University of Colorado, Boulder, United States of America

## Abstract

Primordial germ cells (PGCs) arise elsewhere in the embryo and migrate into developing gonadal ridges during embryonic development. In several model animals, formation and migration patterns of PGCs have been studied, and it is known that these patterns vary. Sturgeons (genus *Acipenser*) have great potential for comparative and evolutionary studies of development. Sturgeons belong to the super class Actinoptergii, and their developmental pattern is similar to that of amphibians, although their phylogenetic position is an out-group to teleost fishes. Here, we reveal an injection technique for sturgeon eggs allowing visualization of germplasm and PGCs. Using this technique, we demonstrate that the PGCs are generated at the vegetal pole of the egg and they migrate on the yolky cell mass toward the gonadal ridge. We also provide evidence showing that PGCs are specified by inheritance of maternally supplied germplasm. Furthermore, we demonstrate that the migratory mechanism is well-conserved between sturgeon and other remotely related teleosts, such as goldfish, by a single PGCs transplantation (SPT) assay. The mode of PGCs specification in sturgeon is similar to that of anurans, but the migration pattern resembles that of teleosts.

## Introduction

The Acipenseriformes, including sturgeons, is the oldest order within the Actinopterygii other than the Polypteriformes. This order is frequently referred to as “living fossils” in the literature. The fossil record of sturgeons dates back to the Upper Cretaceous [1], and mitochondrial DNA analysis suggested that they had diverged from an ancient, pre-Jurassic teleost lineage approximately 300 million years ago (Mya) [2].

Studies of sturgeon embryogenesis provide us a valuable point of comparison for teleosts and amphibians, since their phylogenetic position is that of an out-group of teleosts [3]. Sturgeon eggs have holoblastic cleavage, as in anurans [4]. Development of sturgeon embryos is more similar to *Xenopus* than a teleost, not only in their cleavage pattern but also in many other aspects [5],[6]. Sturgeon embryos form a distinct blastocoel and archenteron, and the neural tube develops from formation of a neural fold and its subsequent closure [7]. In contrast, most teleosts do not show any structure corresponding to a significant segmentation cavity, referred to as a blastocoel and archenteron, and the neural rod is from a neural keel [8].

The yolk volume is a major factor in differences in early embryonic patterning among animals [9]. Vertebrates would have increased their egg size to store yolk during their divergence from ancestral protochordates into fishes. The significant increase in yolk volume of an embryo would have altered the cleavage pattern and germ layer formation. In Actinopterygii, bichirs (*Polypterus*) embryos, which also belong to a phylogenetically ancient group, undergo holoblastic cleavage, similar to amphibians and sturgeons [6],[10]–[12]. In contrast, teleosts embryos such as the zebrafish, belonging to the most modern group of Actinopterygii, undergo meroblastic cleavage; the vegetal half of the egg is the nutritive yolk part that will not differentiate into the embryonic body, while the animal half of the egg, i.e., blastodisc, undergoes cleavage to form three germ layers. Gar (*Lepisosteus*) and bowfin (*Amia*), also ancient species, are considered transition phases from the holoblastic to meroblastic cleavage. They generate giant yolky blastomeres in the vegetal side, and cleavage takes place mostly in the animal hemisphere [13]–[15]. Meroblastic cleavage is thought to be established by the fusion of vegetal blastomeres in holoblastic cleavage, as yolky contents increase during their divarication [9],[12].

**Figure 1:**
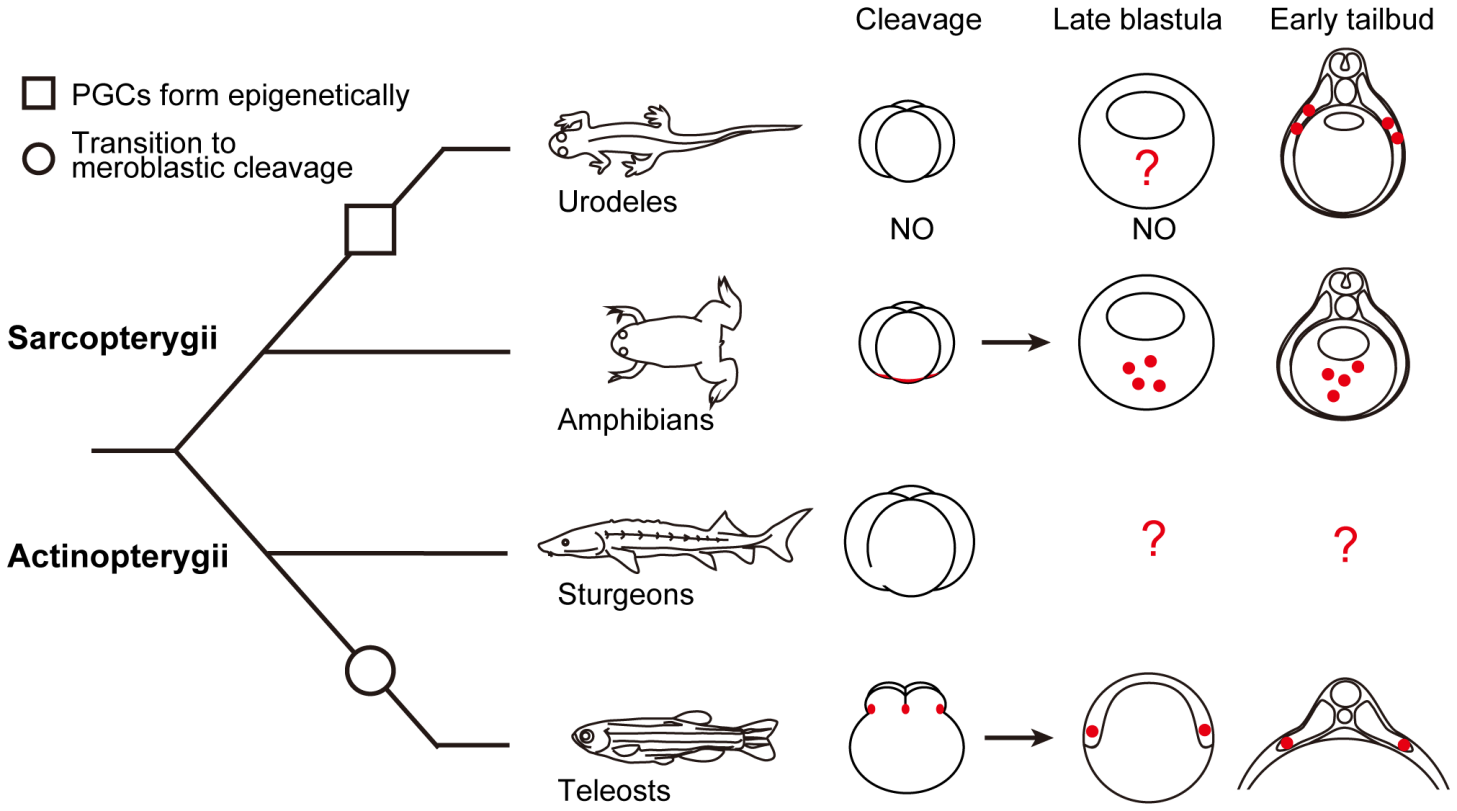
Schematic representation of embryo cleavage patterns, and formation of primordial germ cells (PGCs) and their migration patterns in selected vertebrates. Red area/dots indicate germplasm or the PGCs. NO indicates “not observed”. Note that sturgeons are classified within the Actinopterygii, even though their embryos undergo holoblastic cleavage.

The mode of primordial germ cells (PGCs) formation is altered according to the transition of cleavage from holoblastic to meroblastic (Figure 1). PGCs in anurans and teleosts are specified by inheritance of maternally supplied “germplasm”, a particular region of cytoplasm that specifies the germ-cell fate. In these animals, the germplasm is associated with cleavage furrows [16]–[21]. In *Xenopus*, germplasm containing the “mitochondrial cloud” aggregates in association with the reorganization of microtubules after fertilization [22]. The movement of germplasm can be traced by staining mitochondria with vital dyes during early embryonic development, and the germplasm was reported to aggregate along the forming cleavage furrows [23],[24]. Their aggregation associates directly with the polymerized microtubule network at the vegetal pole [5]. A similar mechanism is involved in the germplasm localization in zebrafish. Here, germplasm becomes enriched at the distal ends of the first and second cleavage furrows and this process requires microtubule function [26],[27]. However, the topology of the germplasm localization is different between *Xenopus* and zebrafish, with localization at the vegetal pole region in *Xenopus*, and at the distal ends of the first and second cleavage furrows of the blastodisc located at the animal hemisphere in zebrafish. In urodeles, also with holoblastic cleavage, PGCs are presumably induced in the primordial ectoderm by cellular interactions between cells located at the vegetal and animal hemispheres during blastula stage [28]. Therefore, the sturgeon is a good model in studying the evolutionary transition of the cleavage pattern from holoblastic to meroblastic division, germplasm localization and the diversification of the determination pattern of PGCs between the inductive and the predetermined (germplasm) mode.

The migration pattern of PGCs toward the gonadal ridge seems to be altered by the position specified for them (Figure 1). In *Xenopus*, the specified PGCs at the vegetal pole region are translocated passively to the center of endoderm, and then they start active migration within the ventral part of the gut endoderm dorsally, and reach the dorsal wall of the body cavity and the gonadal ridge via the dorsal mesentery [16]. It has been reported that SDF-1/CXCR4 chemokine signaling is involved in the directional migration of PGCs toward the genital ridge [29]. Furthermore, contact guidance seems to be involved in this migration to orient the direction of PGCs movement. PGCs adhesion and migration are inhibited by treating the mesentery with anti-fibronectin antibody, suggesting that an orienting fibronectin-containing extracellular matrix is an important factor for PGCs migration in this species [30]. On the other hand, in zebrafish, a relationship between PGCs and the endodermal cells during migration has not been observed, and instead many studies have shown the close association of PGCs with the trunk mesoderm [31]–[33]. PGCs are specified in the marginal region of the blastodisc, with close proximity to the yolk syncytial layer, the presumed mesoendodermal region at the blastula stage, and these cells then migrate dorsally as the embryo develops. During early somitogenesis, these cells migrate along the border of the trunk mesoderm toward the region where the genital ridge will be formed. The whole migration process of the PGCs seems to be organized by the chemo-attractant system, in which CXCR4, CXCR7, and SDF-1a play a main role [34],[35]. In urodeles, PGCs do not migrate within the gut endoderm to reach the gonadal ridge, a pattern relatively similar to that of zebrafish. In the normal embryonic development of urodeles, PGCs arise in the presumptive lateral plate mesoderm and migrate dorsally along with the mesoendodermal interspace and the dorsal mesentery, where these cells then split to the right and left sides and form the gonadal ridge [36]. Only *Xenopus* PGCs use the route of gut endoderm in the first step of their migration to the gonadal ridge.

In sturgeons, the origin of the PGCs and their migratory behavior have not yet been reported; however, the location of the PGCs at 6 days post fertilization (dpf) has been described with conventional and electron microscopy [37],[38]. Sturgeon PGCs would provide us with valuable information to understand gametogenesis, sex differentiation, and evolution of PGCs development in vertebrates. We raise several question about PGCs development in sturgeon (Figure 1): 1) the presence or absence of maternally supplied germplasm in the sturgeon egg, 2) the position where PGCs are specified, and 3) migratory pattern of PGCs. In this study, we took advantage of PGCs transplantation techniques to elucidate whether or not the migratory mechanism of PGCs was conserved between sturgeon and teleosts. Our results clearly show that the specification pattern of the sturgeon PGCs closely resembles that of anurans, and the mechanisms governing migration of PGCs are conserved widely among Actinopterygian fishes.

## Materials And Methods

### Ethics

All experimental procedures were performed in accordance with National and Institutional guidelines on animal experimentation and care, and were approved by the Animal Research Committee of Hokkaido University (approval ID: 22-1) and the Ministry of Agriculture of the Czech Republic (reference number: 53100/2013-MZE-17214).

### Preparation Of Embryos

In this study, we used embryos from pairs of F2 bester {hybrid between beluga (*Huso huso*) and sterlet (*Acipenser ruthenus*) (HY)}, wild-type sterlet (WS), and albino sterlet (AS). HY were kept at the Nanae Fresh-Water Laboratory, Hokkaido University, Japan. WS and AS were kept at the Faculty of Fisheries and Protection of Waters, Research Institute of Fish Culture and Hydrobiology, University of South Bohemia in Ceske Budejovice, Czech Republic. Fish were held in tanks at 13°C. Ovulation and spermiation of HY were induced by an intramuscular injection of 100 µg/kg body weight (b.w.) of the gonadotropin-releasing hormone analogue; des-gly^10^[d-Ala^6^]-LHRH (Sigma). The eggs and sperm were stripped and fertilized one day after injection. To induce spermiation of WS and AS, males were injected by a single intramuscular injection of carp pituitary homogenized extract (CPE) at a dose of 40 mg/kg b.w. Sperm was collected 48 h after hormonal injection by use of a catheter from the urogenital papilla of males, transferred to a separate cell culture container (250 ml), and stored at 4°C until sampling. Ovulation of WS and AS was induced with CPE in two steps: the first, 5 mg/kg b.w. and the second, 45 mg/kg b.w., 12 h after the first injection. The ovulated eggs were collected 18–20 h after the second injection. The eggs were fertilized with sperm in dechlorinated water at 15°C.

Stickiness of the fertilized eggs was removed by treating with 0.04% tannic acid solution or clay water. An outer layer of chorion was removed using forceps in order to manipulate embryos. Embryos were cultured in dechlorinated tap water containing 0.01% penicillin and 0.01% streptomycin at 15°C until hatching. Up to 20 embryos were kept in 100 ml dechlorinated tap water and water was replaced every 24 hours. Embryos from HY were mainly used for GFP-*nos*3 3′UTR mRNA injection for labeling PGCs, FITC-labeling for cell tracing, electron microscopy analysis of unfertilized egg, and PGCs transplantation. Embryos from WS were used for GFP-*nos*3 3′UTR mRNA injection for labeling PGCs and electron microscopy of early cleavage embryos. Embryos from AS were used for observation of GFP aggregation in GFP-*buc* mRNA injected embryos by taking advantage of their unpigmented characteristic. Developmental stages were described as in [7], which describes 45 developmental stages from unfertilized egg to pre-larva. The embryonic staging was determined based on *Acipenser stellatus*, *Acipenser gueldenstaedti*, and *H. huso*. However, early embryonic development of sturgeons is remarkably similar among species and even genera, therefore, the one species’ development is applicable to others, and this information can be generalized [39].

### Microinjection

An artificially synthesized GFP-*nos*3 3′UTR mRNA or GFP-*buc* mRNA was injected into the animal or vegetal pole of the fertilized eggs to investigate the origin of PGCs [40]–[42]. Capped sense mRNAs were synthesized *in vitro* using the mMESSAGE mMACHINE kit (Ambion). The artificially synthesized mRNAs were dissolved in 0.2 M KCl at a concentration of 300 µg/µl. To study the cell lineage, FITC-biotin-dextrans (MW = 10,000) was injected into a single vegetal blastomere at the 64- to 128-cell stage. Labeled cells were analyzed and photographed using a Leica M165 MC imaging system.

### Pgc Transplantation Assay

To study whether the migratory mechanism of PGCs is conserved between Acipenseridae and teleostei, the GFP-labeled PGC of sturgeon was transplanted into a goldfish embryo.

Sturgeon PGCs were visualized with GFP by injecting GFP-*nos*3 3′UTR mRNA at the vegetal pole at 1- to 4-cell stage. After appearance of GFP-positive PGCs around the posterior marginal region of an embryonic body at the late-neural stage, an embryonic fragment containing PGCs was surgically cut from the embryo using fine forceps. The fragment was dissociated into single cells by treating with an enzymatic solution (0.5% citric acid 3Na, 0.1% trypsin, 0.1% collagenase in Ringer’s solution (111.2 mM NaCl, 3.4 mM KCl, 2.7 mM CaCl2 ⋅ 2H2O, 23.7 mM NaHCO3, pH 7.4)). The isolated cells were placed on a glass dish filled with Ringer’s solution containing 3% BSA. A single PGC was aspirated into a glass micro-capillary and transplanted into a goldfish blastula embryo [43]. Endogenous PGCs of host embryos were visualized with red fluorescent protein (RFP) by injecting RFP-*nos*3 3′UTR mRNA at 1- to 4-cell stage. Chimeras were cultured in Ringer’s solution containing 0.01% penicillin and 0.01% streptomycin at 15°C, and observed and photographed under the Leica M165 MC imaging system. The photos taken through filters for GFP and RFP fluorescence were merged into one image using Adobe Photoshop CS5.1.

### Histology And Microscopy

For electron microscopy, unfertilized eggs and embryos at early cleavage stages were fixed with 2.5% glutaraldehyde in PBS, rinsed three times with PBS, and fixed again with osmium tetraoxide for 2 h. Samples were dehydrated through an acetone series, and finally embedded in Spurr’s resin (TAAB Laboratories Equipment Ltd.). Samples were oriented so that sections were cut along the animal-vegital axis. Sections were cut with a diamond knife on a Porter-Blum MT-1 ultramicrotome (Du Pont Sorval) and mounted on formvar-coated slot grids. Sections were stained with uranyl acetate and lead citrate and observed with a JEOL 1011 electron microscope (JEOL).

## Results

### Visualization Of Pgcs By Injecting Gfp-*Nos*3 3′utr Mrna Into The Vegetal Pole Region

**Figure 2:**
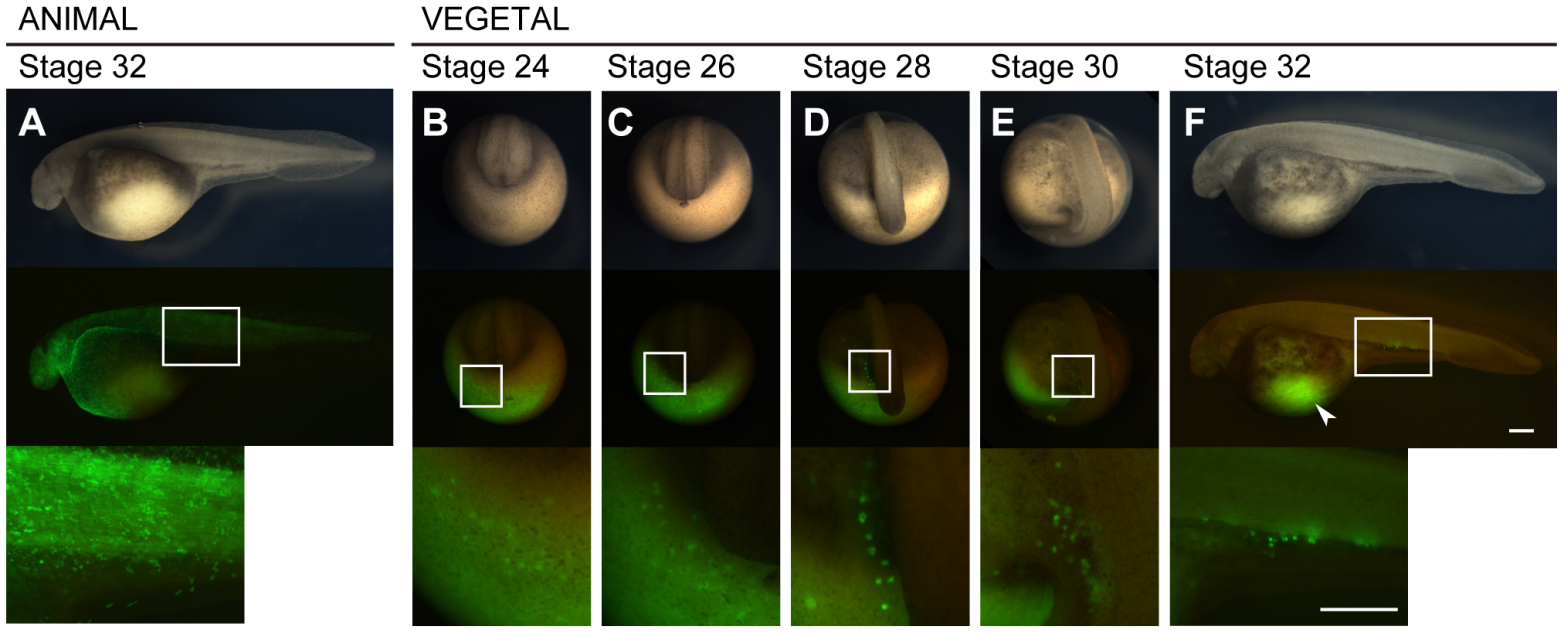
Migration of visualized PGCs in sturgeon embryo. An embryo injected with the GFP-nos3 3′UTR mRNA at the animal pole (A) and at the vegetal pole (B–F). (A) The animal pole labeled embryo at stage 32. (B) The vegetal pole labeled embryo at stage 24. PGCs were initially found around the marginal region of the posterior developing embryonic body at this stage (C) The embryo at stage 26. The PGCs migrated dorsally as the tail rudiment bulged out. Until this stage, the distribution of the labeled PGCs is crescent-like surrounding the developing tail bud. (D) The embryo at stage 28. The PGCs were divided into two populations, at the left- and right-side of the embryonic body. At this stage, fluorescence of PGCs was stronger than during stages 24 and 26. Most PGCs are still localized on the yolk ball. (E) The embryo at stage 30. Most PGCs are located on the yolk extension, but some more ventral were still on the yolk ball. (F) The embryo at stage 32. PGCs are localized at the position where the gonads will develop. Some of these cells migrated axially. Note that PGCs migrated a long distance from their position of origin (arrowhead). The upper, middle, and lower columns indicate bright views, fluorescent views, and magnified fluorescent views of the boxes in the middle column, respectively. B-E are posterior views. E and F are lateral views. The scale bars indicate 500 µm.

To examine the area where PGCs were generated, GFP-*nos*3 3′UTR mRNA, which is known to efficiently visualize teleost PGCs [41], was injected into the animal or vegetal pole region of 1-cell stage embryos. At the blastula stage, GFP expression was observed only at the injected hemisphere. This indicated that the injected mRNA could not diffuse by itself to the opposite hemisphere because of its high molecular weight (about 44,000). In embryos where mRNA was injected into the animal pole, PGCs-like cells were not observed, whereas somatic cells in an embryonic body were labeled with weak GFP expression (Figure 2A, Table 1). On the other hand, in embryos labeled at the vegetal pole, PGC-like cells that had stronger GFP fluorescence than surrounding cells appeared frequently during development (Figure 2B-F; Table 1). In these embryos, weak GFP expression was seen mainly in the ventral yolky position (Figure 2F, arrowhead), while PGC-like cells were observed initially near the tailbud on the yolk-rich endoderm after the closed neural tube stage (Figure 2B–C). These cells detached from the region with weak GFP expression toward the embryonic body. At this stage, these cells distributed in a crescent shape around the developing tail bud (Figure 2C). After that, PGC-like cells continued to move more axially and were split into two populations: left and right side of the embryonic body, as the yolk extension develops during somitogenesis (Figure 2C–E). During this migration, time-lapse imaging showed an active migration of PGC-like cells by the generation of cellular protrusions. At stage 30, PGC-like cells continued to migrate dorsally and most of them settled on both sides of developing yolk cell extensions (Figure 2E). By stage 32, these cells aligned along the border between the embryonic body and the yolk cell extensions (Figure 2F). At this stage, PGC-like cells showed less active behavior for migration, suggesting that they are thereafter moved passively by the surrounding somatic cells. Subsequently, PGC-like cells moved axially on the alimentary canal reaching the dorsal part of the body cavity via the dorsal mesenchyme. An average of 23.5 PGC-like cells were found in each embryo (Table 1). Finally, the PGC-like cells formed two lines of the genital ridge; therefore we concluded that visualized PGCs-like cells are in fact PGCs.

**Table 1:** Efficiency of visualized PGCs in sturgeon at 4 dpf following microinjection of GFP-nos3 3′UTR mRNA or FITC-Biotin-dextran into 1- to 4-cell stage embryos.

	Total no. of embryos	No. of embryos developed normally (%)	No. of embryos with labeled PGC (%)	Average no. of labeled PGC (±SD)	Range
AP*	44	33 (75.0)	0 (0.0)	0.0 (±0.0)	0–0
VP*	47	35 (74.5)	33 (94.3)	23.5 (±17.1)	0–59
FITC	68	33 (48.5)	32 (97.0)	17.0 (±13.0)	0–52
Cont.	42	39 (92.9)	–	–	–

* AP, animal pole; VP, vegetal pole.

### Location Of Pgcs Production

**Figure 3:**
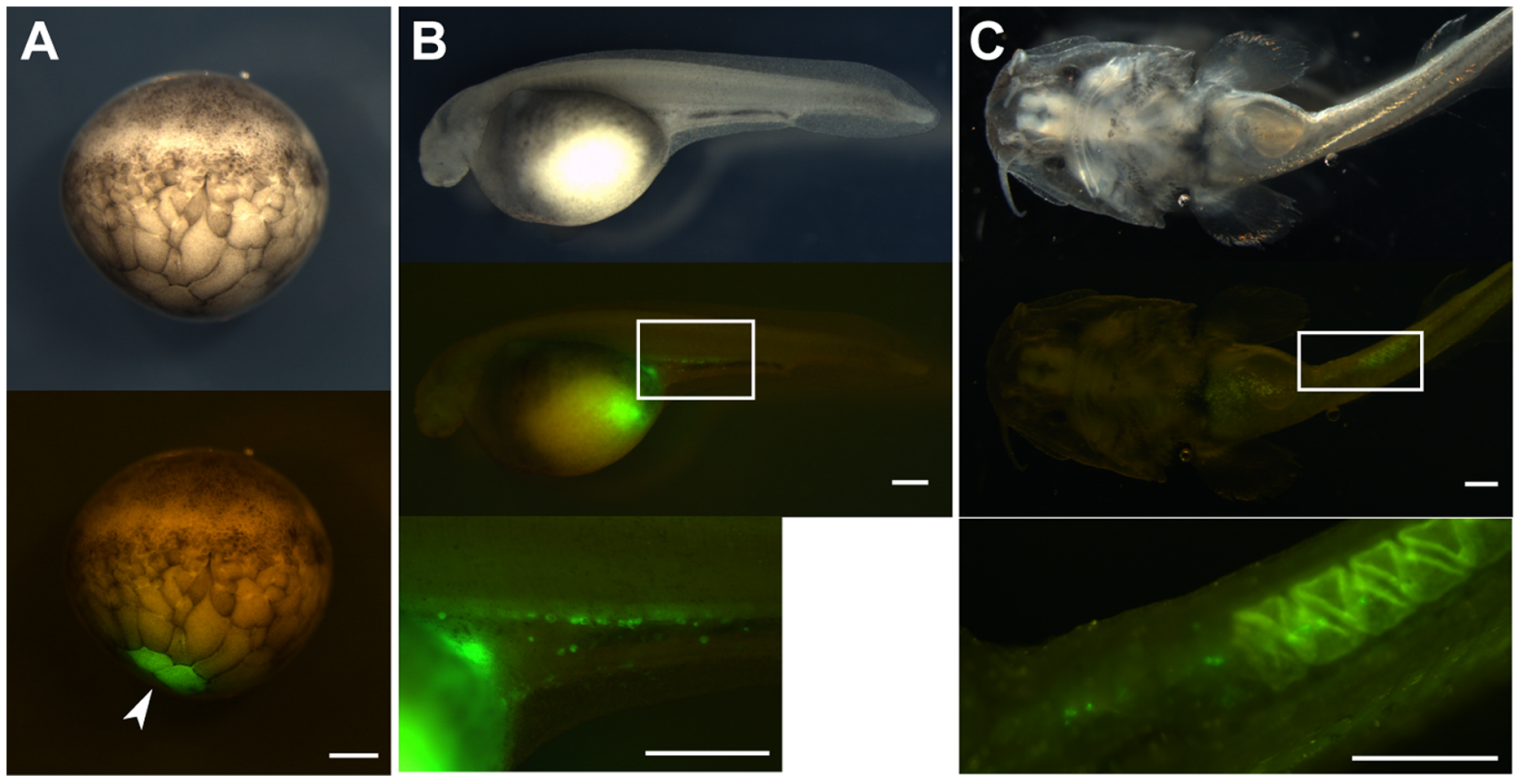
The lineage of a single blastomere at the vegetal pole at blastula stage. (A) The blastula stage embryo with a labeled blastomere with FITC (arrowhead). The upper figure shows the bright view and the lower figure the fluorescent view. (B) The labeled embryo at stage 32. PGCs were located in positions where gonads are expected to develop, apart from the main part of the labeled region. (C) The labeled embryo at 3 weeks post-fertilization. After fry started feeding, PGCs still could be observed at the gonadal ridge of the embryo. The scale bars indicate 500 µm.

By injecting GFP-*nos*3 3′UTR mRNA, we demonstrated that PGCs originate from the vegetal hemisphere. Next, to identify the region of PGCs production, we traced the lineage of a single blastomere located at the vegetal pole at the 64- to 128-cell stage by injecting FITC-dextran (MW; 10,000)(Figure 3A). The FITC-labeled cells largely remained at the ventral position of the embryo at 5 dpf, and it appeared that this location was used as extra-embryonic nutrition after hatching, since FITC-labeled cells were mainly localized within the intestine and the degraded products with FITC were excreted from the excretory pore during development. On the other hand, PGCs were observed as FITC-labeled cells indicating these cells originated from the labeled blastomere. As the embryo develops, FITC-labeled PGCs detached themselves from the main cluster of the FITC-labeled vegetal cells. After that, PGCs migrated toward the yolky extension and localized at the gonadal ridge, as seen in GFP-labeled PGCs (Figure 3B and C). The FITC-labeled PGCs could be observed after hatching. The average number of FITC-labeled PGCs was 17.0 in these embryos (Table 1).

### The Islands Of Germplasm Localized At The Cortical Region Of The Sturgeon Blastula Vegetal Pole

**Figure 4:**
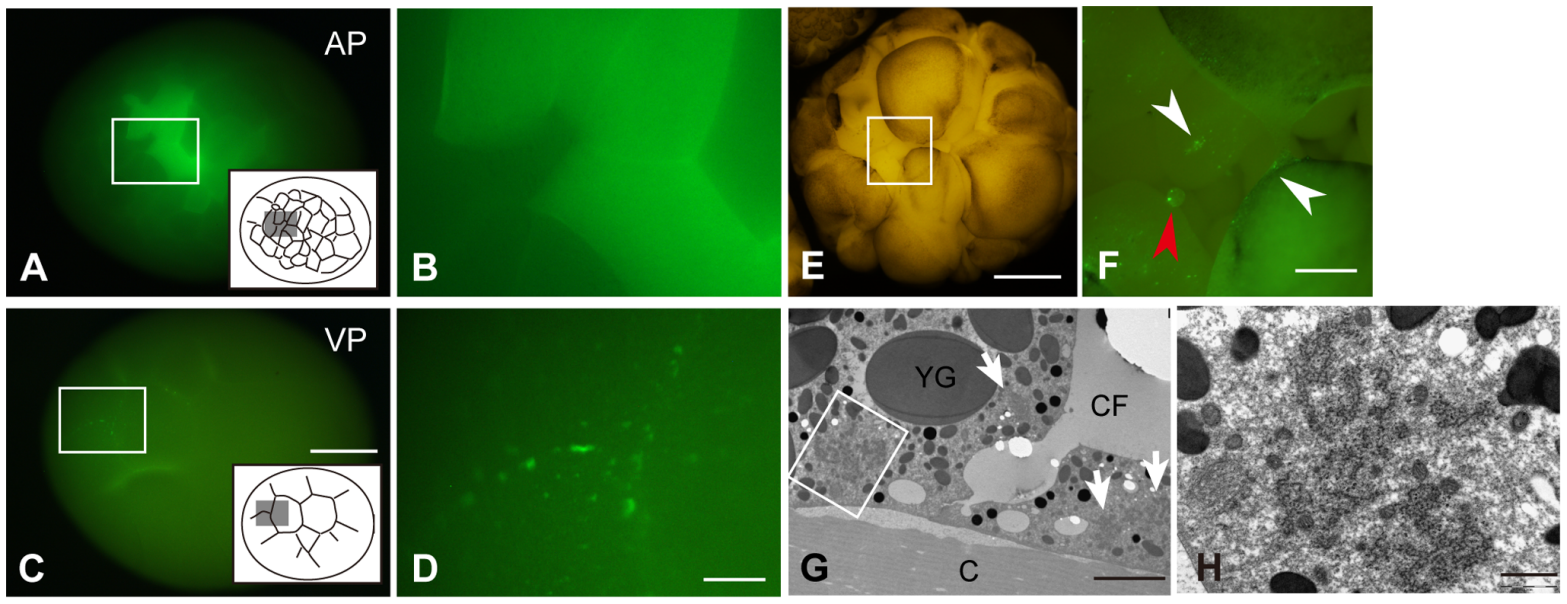
Expression of GFP-Buc protein in an embryo and observation of germplasm with transmission electron microscopy. (A) Expression of GFP-Buc protein at the animal pole. Insert indicates the diagram of this view. (B) The magnified image of the box in (A). (C) Expression of GFP-Buc protein at the vegetal pole. Insert indicates the diagram of this view. Yolky vegetal blastomeres are bigger than those of the animal hemisphere. (D) The magnified image of the box in (C). (E) Vegetal view of a blastula stage embryo injected with GFP-*buc* mRNA at the vegetal pole. Blastomeres were semi-dissociated in order to observe the expression of GFP-Buc protein in the embryo. (F) The magnified and fluorescent image of the boxed area in (E). Arrowheads indicate accumulations of GFP-Buc protein. Note that one accumulation is inherited by a small cell (red arrowhead). (G) Ultrastructure of embryo at the vegetal pole region at 8-cell stage. Mitochondrial cloud and “nuage” were observed near the cleavage furrow (arrows). (H) The magnified image of the box in (G). The “nuage” of the sturgeon appears as distinct amorphous inclusions without the surrounding membrane. This structure is very similar to that is seen in other species [20]. C, Chorion; CF, cleavage furrow; YG, yolk granule. Scale bars in (C) and (E) indicate 500 µm, and 100 µm in (D) and (F); the scale bar in (G) indicates 5 µm, and 1 µm in (H).

We examined whether PGCs are specified by inheritance of germplasm or induction of cellular signaling and then investigated the existence of germplasm in sturgeon. GFP-zf*buc* mRNA was injected into either the animal or vegetal pole of fertilized eggs derived from the albino strain. GFP-zf*buc* protein expressed uniformly at the animal pole at around 32- to 64-cell stage (Figure 4A and B). In contrast, aggregations of GFP-zf*buc* protein were observed at the vegetal pole at the same stage (Figure 4C and D). These aggregations were localized mainly along the cleavage furrows (Figure 4D). Then, to examine the location of GFP-zf*buc* protein aggregations in the vegetal blastomeres at early blastula stage, blastomeres were dissociated. These aggregations were localized at the cortical regions that are contacting neighboring blastomeres, in the inner part of the embryo (Figure 4F, white arrowheads). At the same stage, we observed that some small cells located around the vegetal pole also inherited the aggregation (Figure 4F, red arrowhead). Transmitted electron microscopy (TEM) revealed the presence of a distinct histological structure, similar to “nuage” in an egg. Electron-dense amorphous structures were only found within the cytoplasm in close proximity to the cell membrane around the cleavage furrow, with many mitochondria at the vegetal region (Figure 4G). These structures, however, were not directly attached to the cell membrane, and the distance between them was approximately 0.5–1.0 µm (Figure 4G). These structures were composed of distinct amorphous inclusions with a granular fine structure, lacking a surrounding membrane (Figure 4H). These features were identical to those of the “germplasm” observed in many animal species [16]–[21].

### Mechanism Governing Pgcs Migration Is Conserved Among Actinopterygii Species

**Figure 5:**
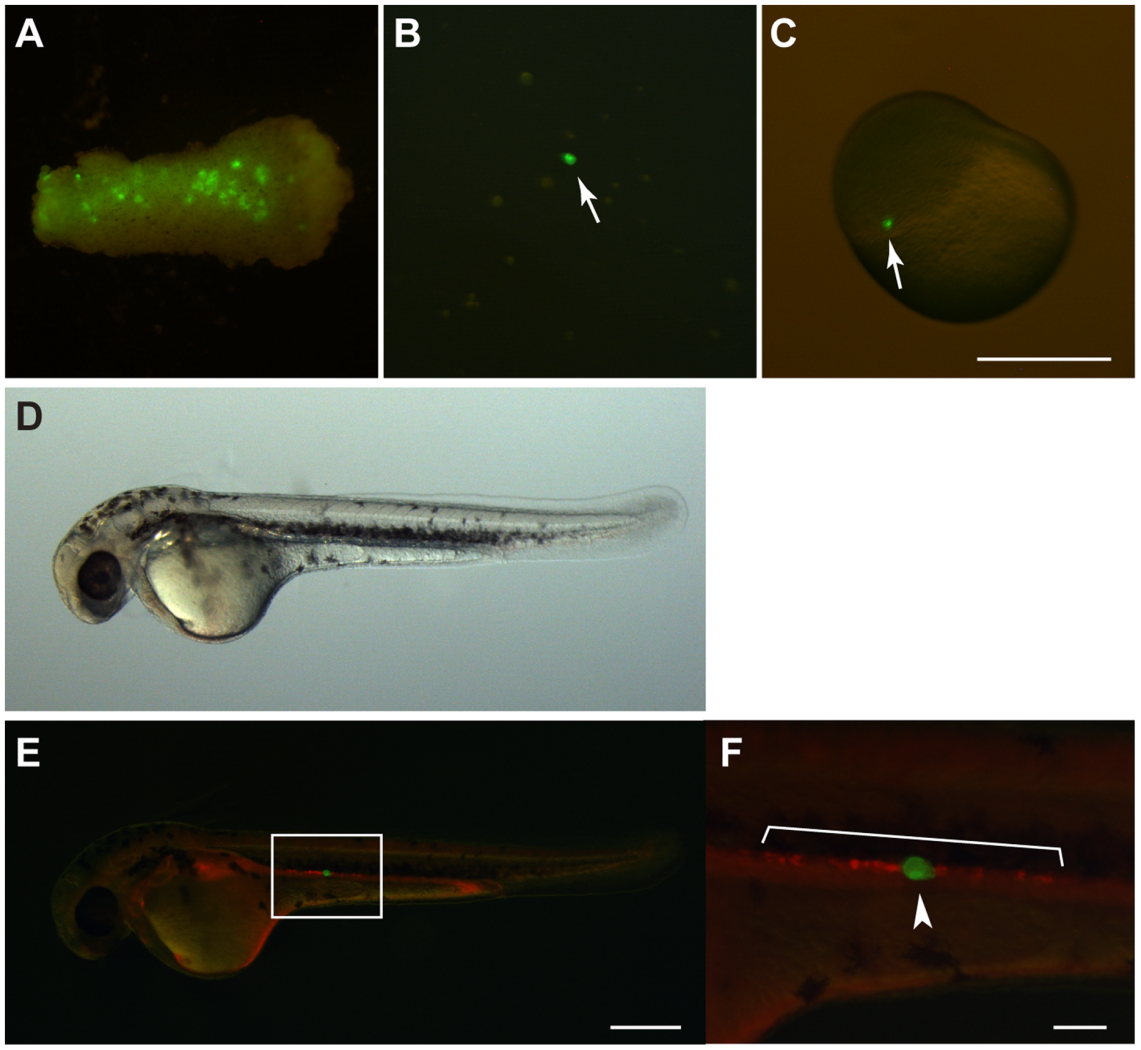
Transplantation of a single PGC from a sturgeon embryo into a goldfish embryo. (A) A removed fragment containing PGCs from sturgeon embryo at stage 26. (B) Dissociated cells from the fragment. Arrow indicates a GFP-labeled PGC. (C) A goldfish embryo at the blastula stage showing the location of an isolated single PGC following transplantation (arrow). (D) A PGC transplanted chimera at 3-dpf. (E) Fluorescence view of (D). (F) The magnified image of the box in (E). RFP-labeled cells indicate endogenous goldfish PGCs and GFP-labeled cell indicates the transplanted sturgeon PGC. Note that the sturgeon PGC localized together with goldfish PGCs at the gonadal region. Scale bars in (C) and (E) indicate 500 µm; the scale bar in (F) indicates 100 µm.

Sturgeon PGCs could be visualized with GFP/FITC fluorescence as described above. One simple and direct approach in studying PGCs migration is an interspecific PGC transplantation and observation of its behavior in the host embryo. It has already been shown that allo- and xenogeneic transplantation of a single visualized PGC from an embryo of one species to that of another will produce a germ-line chimera [43]–[46]. To demonstrate that the migratory mechanism is conserved between sturgeons and teleosts, we transplanted a GFP-labeled sturgeon PGC into a goldfish blastula embryo, in which endogenous PGCs were labeled with RFP by injecting RFP-*nos*3 3′UTR mRNA (Figure 5A–C). Thirty-six chimeras were produced, of which 2 embryos contained a transplanted donor PGC at the gonadal region at 3 dpf (Table 2). The size difference of PGCs between sturgeon and goldfish was clear. Although a significant number of donor PGCs migrated along with a group of host PGCs during early somitogenesis, some of them disappeared before arriving at the gonadal region. Donor-derived PGCs, which localized at the gonadal region, were positioned side by side with host PGCs (Figure 5D–F), indicating that sturgeon PGCs could recognize the guidance signals for goldfish PGCs during migration. However, transplanted PGCs that successfully migrated to the gonadal region disappeared after 4 dpf.

**Table 2:** Migration rate of transplanted sturgeon PGCs toward gonadal region of goldfish embryo.

	Total no. of embryos	No. of embryos developed normally (%)	No. of embryos which have transplanted PGCs	No. of embryos which have transplanted PGCs at gonadal region
Exp. 1	8	7 (87.5)	1 (14.3)	1
Exp. 2	18	15 (83.3)	1 (6.7)	1
Total	26	22 (95.7)	2 (9.1)	2

## Discussion

### Germplasm Is Supplied Maternally And Deposited In The Vegetal Pole Of The Sturgeon Egg

In this study, we showed that PGCs were successfully visualized by injecting the GFP-*nos*3 3′UTR mRNA into the vegetal hemisphere of the sturgeon embryo. Localization of othologs of the *nanos* to germ cells have been found in nearly all taxa studied [47],[48]. It has been previously shown that the 3′UTR of *nanos*3 is subjected to degradation in somatic cells, but is stabilized in PGCs by interaction with the microRNA (miR-430), Dnd, and Dazl [49]–[51]. Our results strongly suggest that the mechanism of conditional regulation of *nanos*3 mRNA should be conserved in sturgeon, as in other teleosts [41],[45]. However, we also observed comparatively high GFP-expression in somatic cells. A similar effect was reported by Saito et al (2006) when GFP-zebrafish *nos3* 3′UTR mRNA was injected into the eggs of phylogenetically distant species [41]. They found that the background expression of GFP in somatic cells was stronger than when the RNA was injected into zebrafish eggs or those of related species. The microRNA miR-430-mediated clearance of maternal transcribed, if it exists in sturgeon, might show relatively weak function on the zebrafish nos3 3′UTR mRNA due to the distant phylogenetic relationship of the two species, whereas in sturgeons, degradation mechanism of maternal transcripts and its inhibition in germ cells are unknown. Also, it is possible that germplasm islands that are not used for PGC formation may protect the injected *nos3* 3′UTR from degradation. Regardless of the explanation for the somewhat high expression in somatic cells, it is worth noting that this expression decreased during embryo development whereas that in PGCs increased. We therefore believe it is reasonable to conclude that the function of *nos3* 3′UTR mRNA is conserved across fish species.

Next, we injected FITC into a single blastomere at the vegetal pole at 64- to 128-cell stage and found that PGCs were produced within the blastomere. In urodeles, in which PGCs are likely induced by interaction between animal and vegetal blastomeres, PGCs are formed in the animal blastomeres (animal cap), instead of the vegetal blastomeres [28]. However, in anurans, PGCs are specified in the presumptive endoderm that is derived from blastomeres located at the vegetal pole [52]. Our result supports the idea that sturgeon PGCs are specified in the vegetal hemisphere around the vegetal pole, as in anurans.

We next conducted GFP-zf*buc* mRNA injection into eggs of the albino strain. Usually sturgeon eggs are heavily pigmented on the cortical region and these pigments block observation of faint GFP signals. By using the albino strain, however, we solved this problem and the GFP fluorescence could be clearly observed. The islands of GFP-zfBuc protein, the mRNA product, were observed when the RNA was injected into the vegetal pole but not the animal pole. The *bucky ball* gene is a well-conserved gene in animals, although the function of the gene and protein as regards germplasm formation has not been well-studied except in zebrafish [42],[53]. In zebrafish, Buc protein has an important role in assembling the germplasm in an oocyte and early stage embryo, and it is possible to visualize developing germplasm in an embryo by injecting chimeric RNA composed of GFP and *buc* ORF, GFP-zf*buc* mRNA [42]. Namely, Buc protein has a germplasm localization signal [42]. The GFP-buc protein aggregations resembled in form zebrafish germplasm [27]. In zebrafish, the small aggregations of germ cell specific RNAs distribute at the animal pole and, as an embryo cleaves, the aggregations form rod-shaped accumulations at the cleavage furrows, as seen in the sturgeon. This result suggests that, in a sturgeon embryo, the mechanism of localization of Buc protein to germplasm is conserved, even though the position where the protein accumulates is different. Furthermore, with electron microscopy, we found that germplasm was composed of “nuage” and mitochondria near the cleavage plane at the same position, where GFP-zfBuc aggregations were observed during early cleavage. The structure of the nuage in the sturgeon egg closely resembled that of *Xenopus laevis* [52]. Similarly, sturgeon nuage is essentially similar to that of zebrafish, goldfish, medaka and goby, although the structure is more abundant in the sturgeon egg than the other teleosts [20],[21]. This is direct evidence that the sturgeon egg has germplasm at the vegetal pole, and it is reasonable to conclude that the GFP-zf*buc* expression should reflect the localization of germplasm in the early embryonic stage. Taken together, these findings strongly suggest that the sturgeon PGCs should be specified by the inheritance of germplasm, which is maternally supplied and deposited at the vegetal pole region of the egg. In fact, Zelazowska et al have reported that “balbiani cytoplasm”, which contains nuage-like structures, was observed in stage I to III oocytes of Russian sturgeon (*Acipenser gueldenstaedtii*) [54]. However, the “balbiani cytoplasm” that they observed was interspersed around the nucleus in the oocytes and accumulation at the vegetal pole was not observed at these stages [54]. How the germplasm localizes at the vegetal pole after stage III remains to be explained.

It is assumed that the epigenetic mode of PGCs specification, in which inductive signals from somatic cells specify germ cells, might be the original pattern in the animal kingdom [47],[55]. Johnson and colleagues hypothesized that induction as seen in urodeles and mammals may be the ancestral pattern of PGCs formation, while predetermined PGCs formation, as seen in teleosts and anurans, may have evolved independently [56]–[58]. According to this hypothesis, the close similarity of the patterns between sturgeon and anurans must be a result of convergent evolution. To confirm this, PGCs development should be analyzed in other animals, such as Sarcopterygii and Lepisosteiformes.

### Migration Of Sturgeon Pgcs

**Figure 6:**
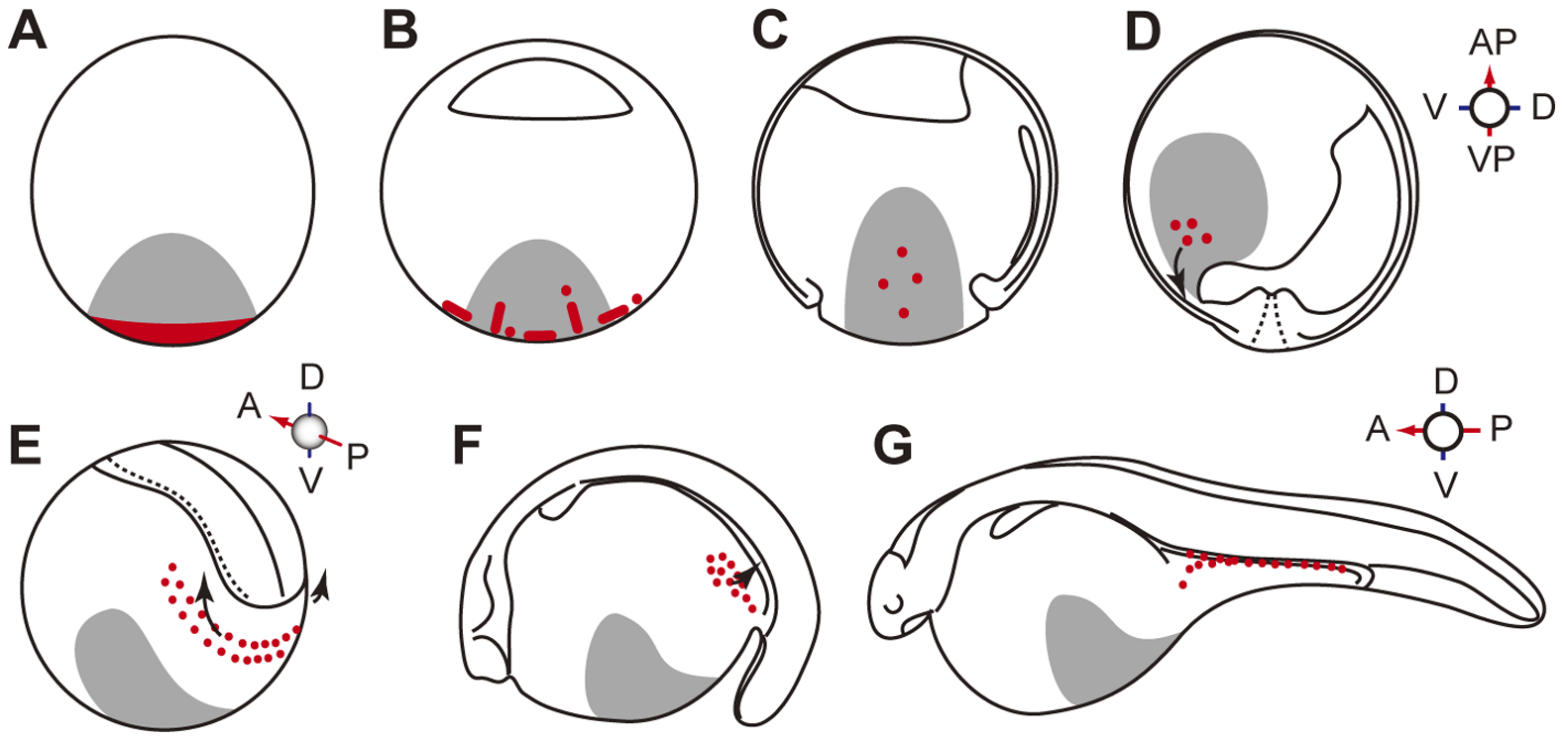
A schematic illustration of development of sturgeon PGCs during embryonic development. (A) 1-cell stage. (B) Early cleavage stage. (C) Gastrula stage: a cross-section cut along animal-vegetal axis. (D) Neurula stage: a cross-section cut along animal-vegetal axis. (E) Closed neural tube stage: posterior lateral view. (F) 4-dpf embryo: lateral view. (G) 5-dpf embryo: lateral view. The regions colored gray show the position labeled by GFP or FITC-dextran injected at the vegetal pole of an early-stage embryo. The red regions in (A) and (B) show the embryonic area where germplasm was observed by TEM. Red dots in (C) and (D) are presumptive PGCs. PGCs were difficult to observe as a single cell, since the PGCs still remained in a group of GFP/FITC positive vegetal cells at this stage. Red dots in (E–G) are PGCs in migration. At stage (E), PGCs showed the crescent-like localization on the yolky cells around the posterior part of the embryonic body. Around stage (E) to (F), PGCs exhibited active movements with the cellular protrusions. After stage (G), however, most PGCs transformed into a round shape and were passively moved toward the gonadal ridge via the mesenchyme.

In our study, we revealed the origin and migration pattern of PGCs in sturgeon (Figure 6). The migratory route of sturgeon PGCs from their formation site to the gonadal ridge differs from that of anurans, although these cells were specified in a similar manner to anurans. In *Xenopus*, PGCs specified at the vegetal pole migrate toward the gonadal ridge within the endoderm [16]. In contrast, sturgeon PGCs migrate after Stage 22 on the yolk ball and the extension toward the gonadal ridge via the mesenchyme. Such a migration pattern resembles that observed in many teleosts, in which PGCs migrate on the yolk ball around the border of the lateral plate mesoderm [41]. In fact, transplanted sturgeon PGCs migrate toward the gonadal ridge of the goldfish, along with the endogenous goldfish PGCs. This result clearly demonstrates that the mechanisms governing migration of PGCs are widely conserved and are common between goldfish and sturgeon, regardless of the considerable differences in embryonic characters, including size, cleavage type, developmental pattern, and the position where PGCs are specified. However, the survival rate of transplanted PGCs was quite low, and most of them disappeared during migration. The PGCs that successfully arrived at the gonadal ridge also disappeared as the embryo developed further. It has been reported that in the chimera produced between closely related species, the transplanted PGCs can contribute to gonadal development and gave rise to functional gametes [43]. However, when transplantation was performed between remotely related fishes from different orders, donor PGCs disappeared during development [45]. Disappearance of the sturgeon PGCs in the goldfish gonad cannot be explained by an immune response, but rather by the loss of maintenance ability of the transplanted germ cells due to the surrounding environment. For example, transplanted PGCs disappeared prior to the morphological and functional differentiation of the thymus gland. The incompatibility between host somatic cells and donor PGCs might result in disappearance of transplanted PGCs in a chimeric animal.

### Fate Of Vegetal Yolky Cells And Pgcs Development In Sturgeon

Germplasm in *Xenopus* concentrates near the vegetal pole by the time the second cleavage furrow reaches there at the 4- to 8-cell stage [17],[22],[23],[59]–[63]. Germplasm is translocated from the surface to inner portions of the embryo along the furrows by cortical ingression [22],[23], so that little remains by 32-cell stage. In contrast, many islands of GFP-Buc remain on the vegetal pole surface of the sturgeon embryo even at the blastula stage. This reduced early translocation may be related to the speed of cell division at the vegetal pole. Compared to *Xenopus*, sturgeon germplasm concentrates along the cleavage furrows, but the furrows arrive at the vegetal pole later and the vegetal cells divide very slowly. An important function of germplasm in *Xenopus* is to repress transcription, thereby preventing PGCs from responding to inductive signals from neighboring cells [64]. Transcriptional repression by germplasm has also been shown in *Drosophila* and *C. elegans* [65], suggesting that this activity is the major role for germplasm in the early embryo. If sturgeon germplasm is widely distributed for a long period at the vegetal hemisphere, how can cells, except for PGCs, avoid transcriptional repression here?

One possibility is that transcriptionally repressed vegetal cells can be tolerated in sturgeon because most vegetal cells serve only a nutritional function and do not contribute to any embryonic differentiation. In the present experiment, the descendants of FITC-labeled blastomere at the 64- to 128-cell stage were localized within the alimentary canal after hatching and were then digested and excreted as a fry develops, except for the PGCs. This result demonstrates that blastomeres at the vegetal pole are used as extra-embryonic tissues exclusively for nutritional function. The developmental fate of the yolky cells (or cell mass) at the vegetal pole is different among animals. In *Xenopus*, vegetal blastomeres differentiate to embryonic endoderm. In contrast, in teleosts, the vegetal yolk cells never differentiate to any embryonic layers, although they supply nutrition and some determinant factors for embryonic patterning and PGCs formation [27],[66]–[68]. The endoderm originates from the marginal zone of the blastodisc. In bichirs, however, which are also known as “ancient fishes”, the yolky cell mass located at the vegetal part of the egg does not develop into endoderm or mesoderm, that is, these cells are extra-embryonic tissues even though the eggs have holoblastic cleavage [12]. According to detailed studies in bichir and lamprey embryos, Takeuchi et al (2009b) hypothesized that the bichir-type holoblastic development, which has the extra-embryonic blastomeres at the vegetal hemisphere, is the ancestral mode. In sturgeons, if the yolky cell mass at the vegetal pole functions just for extra-embryonic nourishment (as in bichirs and lampreys), then sturgeon PGCs should originate from extraembryonic tissue. In *Eleutherodactylus coqui*, which is a direct developing frog with large eggs (3.5 mm dia), vegetal cells are used as nutritional endoderm and it is suggested that PGCs originate from here [69],[70].

Transcriptionally repressive and an increased volume of vegetal cells, however, could lead to difficulties in migration of PGCs from the vegetal pole to their target, the gonadal ridge. A migratory path for PGCs among the huge vegetal cells might be indirect and prolonged. An alternative migratory route for PGCs might involve avoiding vegetal cells, as we have illustrated for zebrafish (above). This might explain the migratory pattern of PGCs in sturgeon, in which PGCs migrate on the yolk ball, avoiding vegetal cells.
